# Multiple imputation inference for multivariate multilevel continuous data with ignorable non-response

**DOI:** 10.1098/rsta.2008.0038

**Published:** 2008-04-11

**Authors:** Recai M. Yucel

**Affiliations:** Department of Epidemiology and Biostatistics, University at Albany, School of Public HealthOne University Place, Room 139, Rensselaer, NY 12144, USA

**Keywords:** missing data, imputation, linear mixed-effects models, complex sample surveys, longitudinal designs, item non-response

## Abstract

Methods specifically targeting missing values in a wide spectrum of statistical analyses are now part of serious statistical thinking due to many advances in computational statistics and increased awareness among sophisticated consumers of statistics. Despite many advances in both theory and applied methods for missing data, missing-data methods in multilevel applications lack equal development. In this paper, I consider a popular inferential tool via multiple imputation in multilevel applications with missing values. I specifically consider missing values occurring arbitrarily at any level of observational units. I use Bayesian arguments for drawing multiple imputations from the underlying (posterior) predictive distribution of missing data. Multivariate extensions of well-known mixed-effects models form the basis for simulating the posterior predictive distribution, hence creating the multiple imputations. The discussion of these topics is demonstrated in an application assessing correlates to unmet need for mental health care among children with special health care needs.

## 1. Introduction

Scientific enquiry in many fields including social, behavioural and medical sciences aims to analyse observational units with special structures dictating the appropriate statistical techniques. Structures where the observational units are clustered within naturally occurring groups, for example, are usually handled via analyses based on multilevel models. This paper considers additional challenges resulting from missing values in such data structures. In health services research, assessing the quality of received services, for example, observational units such as patients, may be nested within doctors, which are further nested within hospitals. A substantive goal might be investigated using a multilevel model on the received health service as a function of characteristics that are at the level of a patient, doctor and hospital. Such models provide many advantages such as decomposing variance as well as accurate calculation of standard errors.

Although the mechanics and theory of these models are now well understood by the greater scientific community (starting with the pioneering work of Laird & Ware (1982) and others’ work on the dissemination of the underlying computational techniques through software), handling missing values has not been equally well disseminated. In multilevel structures, ‘incompleteness’ can easily become a complex problem and potentially diminishes the validity of the inferences if no statistically sound action is taken. Incompleteness can occur at any level of observational units; in our running example, variables measured at the level of the patient, doctor or hospital may be incompletely observed. Ignoring missing values can lead to inaccurate estimation of important relationships and accuracy measures (Rubin 1987; Schafer 1997; Schafer & Yucel 2002).

Throughout this paper, I will use the following convention in referring to the observational units in multilevel structures. ‘Top-level’ units will correspond to the observational units at the highest level of hierarchy. In our example, this would be patient-level data. Level 1 will refer to the lowest level units, for example, hospitals; level 2 will refer to the units within level 1 units, for example, clinics within hospitals.

### (a) Strategies for handling missing values

Any statistical method used to analyse an incomplete dataset adapts, explicitly or implicitly, a strategy for handling missing values. When no action is taken for dealing with missing values, this can be regarded as ‘case deletion’. Case deletion requires very strict assumptions on the missingness mechanism and representativeness of the remainder of the sample. It is often not plausible especially in multilevel settings (Gelman & Hill 2007, ch. 25). Other strategies for dealing with missing values include regression imputation, matching, etc. Implementation of these methods is often very impracticable when the data include arbitrary and complex missingness as usually is the case in multilevel applications.

In general, there are two preferred approaches when dealing with missing values in multilevel applications: likelihood-based and Bayesian methods. Both approaches share common goals of not systematically biasing or misleading the conclusions of the subject matter enquiry. Likelihood-based methods often use expectation-maximization (EM)-type algorithms and/or other numerical techniques such as Fisher scoring to draw inference by maximum-likelihood estimation. Typically, Bayesian methods are used to draw inference by multiple imputation (MI) and they often employ Monte Carlo techniques to simulate an intractable posterior predictive distribution of missing values.

Likelihood-based methods usually involve a maximization of the likelihood function derived from the underlying model. In missing-data applications, the likelihood function to be maximized is based on the observed data only. Under the ignorability assumption (see §2*a*), this likelihood may be written as

(1.1) $$L(\theta |Y_{\text{obs}})=\int L(\theta |Y_{\text{obs}},Y_{\text{mis}})\;\text{d}Y_{\text{mis}},$$

where *θ* represents the unknown parameters of the model; *Y*_obs_ and *Y*_mis_ denote the observed and missing data, respectively; and *L*(*θ*|*Y*_obs_,*Y*_mis_) denotes the likelihood function based on complete data. EM-type algorithms bring a practical solution to this problem by repeatedly ‘filling in’ missing components of the complete-data sufficient statistics and solving the complete-data problem. The resulting algorithms are simple and stable (i.e. guaranteed to increase the likelihood at each step) but may converge slowly. Algorithms such as Newton–Raphson or Fisher scoring are typically faster to converge.

The maximization of *L*(*θ*|*Y*_obs_) often tends to be quite complicated in multilevel settings, requiring complicated numerical techniques or approximations. Owing to its intense computational and problem-specific nature, model-fitting algorithms have not been developed beyond two-level multivariate-response settings. Shah *et al*. (1997) developed a conventional EM algorithm for the bivariate case. Schafer & Yucel (2002) extended their algorithm via the hybrid of EM and Fisher-scoring algorithm for multivariate linear mixed-effects models for ignorable missingness, improving the computational efficiency. Their method was later implemented as an R package called mlmmm (Yucel 2007).

Another popular method for analysing incomplete datasets is inference via MI (Rubin 1987). In MI, missing data are treated as an explicit source of random variability to be averaged over; this averaging is carried out by simulation. In the complex problems, the process of creating imputations usually involves Markov chain Monte Carlo (MCMC) techniques such as the Gibbs sampler and the Metropolis–Hastings algorithm. To produce the imputations, some assumptions about the data (typically a parametric model) and the mechanism producing missing data need to be made. The assumed data model should be plausible and should be somewhat related to the analyst's investigation (Meng 1994; Schafer 2003). For example, in applications to multilevel data, the model should be capable of preserving correlations arising from the multilevel structure. Despite its growing applications to missing-data problems or even to missing-data-like problems (Reiter & Raghunathan 2007), relatively limited progress has been made in multilevel applications. Several studies (Liu *et al*. 2000; Schafer & Yucel 2002) use multivariate extensions of the well-known linear mixed-effects models in the imputation process. A general overview and current state of MI are given by Harel & Zhou (2007).

In any missing-data problem, it may be possible to handle the missing values either by maximizing the likelihood (1.1) or by MI. Inference by MI may have some practical advantages. MI provides complete datasets for subsequent analyses, allowing the analysts to use their favourite models and software. Another possible reason for preferring MI is that there are many problems where there is no algorithm or procedure available to maximize the likelihood (1.1). In multilevel applications where missing values arbitrarily occur among continuous and/or near-continuous variables on possibly every level of hierarchy, algorithms working with (1.1) lead to computationally infeasible algorithms. Further, the model leading to (1.1) may not be of interest as it typically models variables subject to missing values rather than the variables of the scientific enquiry.^1^ Finally, because MI treats missing data as an explicit source of variation, it is capable of distinguishing ordinary sampling variability from missing-data uncertainty.

The remainder of this paper focuses on imputation models for normal or near-normal variables in multilevel settings. I primarily focus on multivariate extensions of widely used models in multilevel applications and tailoring them to create MI of missing values in the variables observed at any level of hierarchy (i.e. level 1, 2 or 3). The organization of the rest of the paper is as follows: §2 describes the notational convention followed in this paper as well as the models used to produce the imputations (imputation models). Section 3 presents a brief overview of the computational algorithms. In §4, I present a data example where arbitrary missingness on both level 1 and 2 variables presented a challenge in a study assessing correlates to the unmet need for mental health care among children with special health care needs. Section 5 provides a discussion and current as well as future research topics.

## 2. Notation and models

In multilevel studies, missing values may occur at any level of observational units. Our notation will distinguish between the levels of the hierarchy in the following way. Lower case *y* will denote the realized value of a random variable *Y* that is subject to missingness, superscript L_*i*_ will indicate the level of observational unit and the subscripts *i, j* and *k* will indicate the data points within these levels. For example, in a three-level study, incompletely observed set characteristics of patient *k* nested within hospital *j* nested within state *i* will be denoted by $y_{ijk}^{{\text{L}}_{3}}$. $Y_{1}^{{\text{L}}_{3}},\ldots ,Y_{r_{{\text{L}}_{3}}}^{{\text{L}}_{3}}$ will indicate a set of $r_{{\text{L}}_{3}}$ random variables measured for the level 3 units. Similarly, $y_{ij}^{{\text{L}}_{2}}$ will indicate the realized value of a random variable measured at the level 2 unit, for example, hospital *j* within state *i*, and $Y_{1}^{{\text{L}}_{2}},\ldots ,Y_{r_{{\text{L}}_{2}}}^{{\text{L}}_{2}}$ will indicate a set of $r_{{\text{L}}_{2}}$ variables. Finally, $y_{i}^{{\text{L}}_{1}}$ will indicate a set of incompletely observed characteristics of state *i* and $Y_{1}^{{\text{L}}_{1}},\ldots ,Y_{r_{{\text{L}}_{1}}}^{{\text{L}}_{1}}$ will indicate a set of $r_{{\text{L}}_{1}}$ variables at level 1. This presentation can easily be generalized to applications where the indices correspond to non-nested units; or to applications where there are more nestings. In this paper, I only focus on the applications with natural hierarchies in the observational units.

In matrix notation, let $y_{ij}^{{\text{L}}_{3}}$ denote an $n_{ij}\times r_{{\text{L}}_{3}}$ matrix of measurements on characteristics $Y_{1}^{{\text{L}}_{3}},\ldots ,Y_{r_{{\text{L}}_{3}}}^{{\text{L}}_{3}}$, where *n*_*ij*_ are level 3 units nested within level 1 and 2 units. In our running example, *y*_*ij*_ may include health service indicators such as medical expenditure, special health care need indicator of patients nested within doctors nested further within hospitals. The observed and missing portions of $Y^{{\text{L}}_{3}}=(y_{11}^{{\text{L}}_{3}},\ldots ,y_{M,n_{M}}^{{\text{L}}_{3}})$ will be denoted by $Y_{\text{obs}}^{{\text{L}}_{3}}=(y_{11(\text{obs})}^{{\text{L}}_{3}},y_{12(\text{obs})}^{{\text{L}}_{3}},\ldots ,y_{M,n_{M}(\text{obs})}^{{\text{L}}_{3}})$ and $Y_{\text{mis}}^{{\text{L}}_{3}}=(y_{11(\text{mis})}^{{\text{L}}_{3}},y_{12(\text{mis})}^{{\text{L}}_{3}},\ldots ,y_{M,n_{M}(\text{mis})}^{{\text{L}}_{3}})$, respectively. Similarly, let $y_{i}^{{\text{L}}_{2}}$ denote an $n_{i}\times r_{{\text{L}}_{2}}$ matrix of measurements on $Y_{1}^{{\text{L}}_{2}},\ldots ,Y_{r_{{\text{L}}_{2}}}^{{\text{L}}_{2}}$ and $y^{{\text{L}}_{1}}$ denote an $M\times r_{{\text{L}}_{1}}$ matrix of measurements on $Y_{1}^{{\text{L}}_{1}},\ldots ,Y_{r_{{\text{L}}_{1}}}^{{\text{L}}_{1}}$. I will allow portions of

$$Y^{{\text{L}}_{2}}=(y_{1}^{{\text{L}}_{2}},\ldots ,y_{\sum_{i=1}^{M}M_{i}}^{{\text{L}}_{2}}),$$

where $\sum_{i=1}^{M}M_{i}$ is the total number of level 2 units, and $Y^{{\text{L}}_{1}}$ (matrix of $M\times r_{{\text{L}}_{1}}$) to be ignorably missing, and let $\left(Y_{\text{obs}}^{{\text{L}}_{2}},Y_{\text{mis}}^{{\text{L}}_{2}}\right)$ and $\left(Y_{\text{obs}}^{{\text{L}}_{1}},Y_{\text{mis}}^{{\text{L}}_{1}}\right)$ denote the observed and missing portions of the measurements for the level 2 and 1 units, respectively. Completely observed auxiliary data on any observational units at level 1, 2 or 3 will be denoted by *X*, *X*_*i*_ or *X*_*ij*_, respectively.

The final set of notation is given to define ‘missingness mechanisms’. I will let $R^{{\text{L}}_{i}}$ denote a matrix of indicator variables whose elements are 0 or 1 identifying whether the elements of $Y^{{\text{L}}_{i}}$ are missing or observed. The dimension of $R^{{\text{L}}_{i}}$ is the same as that of $Y^{{\text{L}}_{i}}$.

### (a) Common models on missingness mechanism in multilevel applications

Statistical methods adopted to deal with missing values ranging from case deletion to model-based MI assume a mechanism that leads to missing values in the underlying dataset. Some techniques explicitly state this mechanism while others state it rather implicitly. Most of the methods commonly practised such as those implemented in software products (pan: Schafer & Yucel 2002; MLWIN: Rasbash *et al*. 2006; or others for cross-sectional data such as NORM: Schafer 2000; IveWare: Raghunathan *et al*. 2001; PROC MI: SAS Institute 2001) assume that missing values are missing at random (MAR; Rubin 1976). Below I assume that the missingness is only at level 3 units.

The missing values are said to be MAR if $P(R_{ijk}^{{\text{L}}_{3}}|Y_{\text{obs}}^{{\text{L}}_{3}}=y_{\text{obs}}^{{\text{L}}_{3}},Y_{\text{mis}}^{{\text{L}}_{3}},\theta^{{\text{L}}_{3}})=P(R_{ijk}^{{\text{L}}_{3}}=r_{ijk}^{{\text{L}}_{3}}|Y_{\text{obs}}^{{\text{L}}_{3}}=y_{\text{obs}}^{{\text{L}}_{3}},\theta^{{\text{L}}_{3}})$ holds for all $\theta^{{\text{L}}_{3}}$, where $\theta^{{\text{L}}_{3}}$ contains all unknowns of the model at level 3, and all *i*, *j*, *k*, where *i* indexes the cluster and *j* and *k* index the data points within cluster *i*. This assumption states that the probability distribution of the missingness indicators may depend on the observed data but not on the missing values. A special case of MAR is missing completely at random (MCAR) in which $P(R_{ijk}^{{\text{L}}_{3}}|Y_{\text{obs}}^{{\text{L}}_{3}}=y_{\text{obs}}^{{\text{L}}_{3}},Y_{\text{mis}}^{{\text{L}}_{3}},\theta^{{\text{L}}_{3}})=P(R_{ijk}^{{\text{L}}_{3}}|\theta^{{\text{L}}_{3}})$ for all $\theta^{{\text{L}}_{3}}$. In MCAR, the probability distribution of missingness is independent of both the observed and missing data. Finally, if MAR is violated, the probability distribution depends on the missing values and the missingness mechanism is said to be missing not at random (MNAR). In the case of MNAR, a joint probability model must be assumed for the complete data as well as the *R*_*ij*_, the missingness indicators. Most of the software packages performing MI inference rely on MAR (e.g. SAS PROC MI, MLWIN or R pan package). The models presented here assume that the missingness mechanism is ignorable in the sense defined by Rubin (1976), that is, the missing data are MAR and the parameters of the missingness distribution and the complete-data distribution are distinct (see more detailed discussion in Rubin 1976 and Schafer 1997). The ‘ignorability’ merely means that the missingness mechanism can be ignored when performing statistical analyses; in other words, no harm is done working with the observed data. This should not be understood as discarding any missing datum: it should be understood that working with the observed likelihood $L(\theta |Y_{\text{obs}})=\int P(Y_{\text{obs}},Y_{\text{mis}};\theta )\;\text{d}Y_{\text{mis}}$ is the same as working with the full likelihood for *θ*.

The literature has been somewhat hesitant in identifying a potential MNAR mechanism on multilevel survey applications. A simple case occurs when the response probability is not fully explained by observed characteristics and varies by level 2 and 1 observational units. It is possible that what motivates the inclusion of random effects into the substantive model motivates the similar cluster-specific or latent effects to be a factor in the missingness mechanism. One of the main motivations for arguing for a rich imputation model is to capture as much cluster variation as possible in the response mechanism. The purpose of a rich imputation model is not to draw inferences on the underlying population, but rather to obtain plausible imputations while accounting for the data structure and reasons for missingness.

### (b) Imputation models

Below I propose models used to jointly impute missing values in the variables measured at any level in a three-level study. Extensions to higher levels are trivial. This section provides the essence of these models in terms of building imputation models and considers distinct models used to derive the underlying posterior predictive distribution of missing values. Specification of prior distributions in these models is discussed in §2*b*(i).

#### (i) Level 3 imputation model

Suppose $Y_{1}^{{\text{L}}_{3}},\ldots ,Y_{r}^{{\text{L}}_{3}}$ denote a set of continuous variables subject to missing values. A version of a joint model that generalizes a well-known linear mixed model to multivariate responses is given by

(2.1) $$y_{ij}^{{\text{L}}_{3}}=X_{ij}\beta +Z_{ij}b_{i}+W_{ij}c_{ij}+\epsilon_{ij},$$

where *i*=1, 2, …, *M*; *j*=1, 2, …, *M*_*i*_; vec(*b*_*i*_)∼*N*(0, *Ψ*); vec(*c*_*ij*_)∼*N*(0, *Γ*); and $\text{vec}(\epsilon_{ij})∼N(0,\Sigma ⊗I_{n_{ij}})$. The *fully observed* covariate matrices *X*_*ij*_(*n*_*ij*_×*p*), *Z*_*ij*_(*n*_*ij*_×*q*_1_) and *W*_*ij*_(*n*_*ij*_×*q*_2_) correspond to the fixed effects *β* and the first- and second-level random effects *b*_*i*_ and *c*_*ij*_, respectively. I assume only that $\beta \in R^{pr}$, *Σ*>0, *ψ*>0 and *Γ*>0. A block diagonal structure can be assumed for *ψ* and/or *Γ* depending on the application.

The choice between block diagonal versus unstructured depends on the application. When modelling a large number of variables subject to missingness, it may be advantageous to restrict *ψ* and/or *Γ* to a block diagonal structure. A block diagonal version would only indicate that the underlying random effects are assumed to be independent *a priori* and this independence would not necessarily hold in their perspective posterior distributions.

Note that what should be included in *X*_*ij*_ is driven by (i) making assumptions on the missingness mechanism plausible by enriching the imputation model to incorporate correlates of missing variables, (ii) finding the relationships that should be reflected in the subsequent analyses so that biases do not occur, and (iii) improving the prediction of the missing values.

It is important to note that the model (2.1) is merely used to impute missing values; the meaning or interpretation of its parameters is not of primary interest. A sensible imputation method for multivariate longitudinal or clustered data should preserve the basic relationships among the variables and the correlations among observations from the same subject or cluster. The model (2.1) is capable of preserving these effects while it simulates missing values in an acceptable manner. In many cases, post-imputation analyses will be based on less elaborate models, for example, a model for one response variable given the others. In other cases, effective analyses may be carried out under a model somewhat different from that used to impute missing values. The performance of MI when the imputer's and analyst's models differ was addressed by Meng (1994), Rubin (1996) and Schafer (2003). In practice, inference by MI is fairly robust to departures from the imputation model because that model effectively applies not to the entire dataset but only to its missing parts. For binary or ordinal variables, one can use the model (2.1) as an approximation and round off the imputed values to the nearest category, as commonly practised. Simulations have shown that the biases incurred by such rounding procedures may be minor (Schafer 1997; Horton *et al*. 2003; Bernaards *et al*. 2006; Demirtas 2008). Methods tailored specifically towards binary responses are proposed by Yucel *et al*. (2008), and Demirtas & Hedeker (2007). A more principled but complicated approach may involve introducing random effects into the general location model for multivariate data with continuous and categorical variables (Olkin & Tate 1961; Schafer 1997).

#### (ii) Level 2 imputation model

Now consider a dataset consisting of level 2 units (total of $\sum_{i=1}^{M}M_{i}$) for which a set of variables $Y_{1}^{{\text{L}}_{2}},\ldots ,Y_{r_{{\text{L}}_{2}}}^{{\text{L}}_{2}}$ are incompletely observed. In our example, these variables describe doctor characteristics within hospitals. As described in §2*a*, $y_{i}^{\text{L}2}$ denotes a matrix of dimension $M_{i}\times r_{{\text{L}}_{2}}$ containing measurements $Y_{1}^{{\text{L}}_{2}},\ldots ,Y_{r_{{\text{L}}_{2}}}^{{\text{L}}_{2}}$ on *M*_*i*_ doctors within hospital *i*. The observed patient-level characteristics can be used in an auxiliary sense by aggregating patients’ (imputed or observed) values to a doctor level:

(2.2) $$y_{i}^{{\text{L}}_{2}}=X_{i}\beta +Z_{i}b_{i}+\epsilon_{i},$$

where i=1,2,…,M; $\text{vec}(b_{i})∼N(0,\Psi )$; and $\text{vec}(\epsilon_{i})∼N(0,\Sigma ⊗I_{M_{i}})$. The fully observed covariate matrices *X*_*i*_(*M*_*i*_×*p*) and *Z*_*i*_(*M*_*i*_×*q*) correspond to the fixed effects *β* and the first-level random effects *b*_*i*_. I assume only that $\beta \in R^{pr}$, *Σ*>0 and *Ψ*>0. Similar to the imputation model for level 3, a block diagonal structure can be assumed for *Ψ* depending on the application.

#### (iii) Level 1 imputation model

Suppose that a set of variables $Y_{1}^{{\text{L}}_{1}},\ldots ,Y_{r_{{\text{L}}_{1}}}^{{\text{L}}_{1}}$ are incompletely observed for a set of *M* level 1 units. There are many well-established computational techniques that one can adapt to draw imputations under an assumed model. Here we consider fully parametric approaches and allow different models depending on the nature of the variables. I adapt imputation routines described by Schafer (1997) for imputing missing values among the observations of independently selected level 1 units. These routines use one of the following models depending on the variable types:

- Multivariate normal distribution is used to impute *continuous* $Y_{1}^{{\text{L}}_{1}},\ldots ,Y_{r_{{\text{L}}_{1}}}^{{\text{L}}_{1}}$.
- Loglinear model (usually saturated) is used to impute *categorical* $Y_{1}^{{\text{L}}_{1}},\ldots ,Y_{r_{{\text{L}}_{1}}}^{{\text{L}}_{1}}$.
- A general location model (Olkin & Tate 1961) is used to the imputed *mixture of continuous and categorical* $Y_{1}^{{\text{L}}_{1}},\ldots ,Y_{r_{{\text{L}}_{1}}}^{{\text{L}}_{1}}$.

#### (iv) Prior distributions

It is known that, in mixed-effects models, improper prior distributions for the covariance components may lead to computational problems in Monte Carlo simulations due to non-existent posterior distributions. For this reason, proper prior distributions for the covariance matrices are highly recommended. For simplicity, I apply independent inverted Wishart priors on the variance components $\Sigma^{-1}∼W(\nu_{1},\Lambda_{1})$ and $\Psi^{-1}∼W(\nu_{2},\Lambda_{2})$, where *W*(*ν*, *Λ*) denotes a Wishart variate with *ν*>0 degrees of freedom and mean *νΛ*>0. This prior is appropriate for a model with unstructured *Ψ*.

Consider the level 3 imputation model and the choices of priors on the variance–covariance matrix *Γ*. When modelling a large number of response variables at once, it may be advantageous to restrict *Γ* (and/or *Ψ*) to a block diagonal structure—not only for the purpose of obtaining prior guesses but also for the ease of computations. If *Γ* is block diagonal, then independent inverted Wishart prior distributions may be applied to the *q*×*q* non-zero blocks, $\Gamma_{j}^{-1}∼W(\nu_{j},\Lambda_{j})$ for *j*=1, 2, …, *r*. Weak priors are obtained by setting *ν*_*j*_=*q* and $\Lambda_{j}^{-1}=\nu_{j}{\hat{\Gamma }}_{j}$, where ${\hat{\Gamma }}_{j}$ is an estimate or prior guess for *Γ*_*j*_. The distributions for these blocks become $\Gamma_{j}^{-1}∼W(\nu_{j}^{\prime },\Lambda_{j}^{\prime })$, where $\nu_{j}^{\prime }=\nu_{j}+m$; $\Lambda_{j}^{\prime -1}=\Lambda_{j}^{-1}+\sum_{i=1}^{m}\;c_{ijk}c_{ijk}^{T}$; and *c*_*ijk*_ is the *k*th column of $c_{ij}$.

These priors exist provided that *Λ*_1_>0, *Λ*_2_>0, *ν*_1_≥*r* and *ν*_2_≥*qr*. In choosing values for the hyperparameters, it is helpful to regard $\nu_{1}^{-1}\Lambda_{1}^{-1}$ and $\nu_{2}^{-1}\Lambda_{2}^{-1}$ as prior guesses for *Σ* and *Ψ*, respectively. Small values for *ν*_1_ and *ν*_2_ make the prior densities relatively diffuse, reducing their impact on the final inferences. For *β*, I use an improper uniform ‘density’ over $R^{pr}$.

## 3. Computational algorithms

Missing data $Y_{\text{mis}}=(Y_{\text{mis}}^{{\text{L}}_{1}},Y_{\text{mis}}^{{\text{L}}_{2}},Y_{\text{mis}}^{{\text{L}}_{3}})$ and parameters defining the models stated above $\theta^{{\text{L}}_{3}}=(\beta^{{\text{L}}_{3}},\Psi^{{\text{L}}_{3}},\Gamma^{{\text{L}}_{3}},\Sigma^{{\text{L}}_{3}})$, $\theta^{{\text{L}}_{2}}=(\beta^{{\text{L}}_{2}},\Psi^{{\text{L}}_{2}},\Sigma^{{\text{L}}_{2}})$ and $\theta^{{\text{L}}_{1}}$ are drawn from their posterior distributions using MCMC techniques (Gelfand & Smith 1990; Smith & Roberts 1993; Tanner 1993; Gilks & Spiegelhalter 1996). My computational algorithm consists of three distinct but interrelated Gibbs samplers, a version of the MCMC technique. The ultimate goal is to generate *K* independent draws from a posterior predictive distribution for the missing data

(3.1) $$P(Y_{\text{mis}}|Y_{\text{obs}})=\int P(Y_{\text{mis}}|Y_{\text{obs}},\theta )P(\theta |Y_{\text{obs}})\;\text{d}\theta ,$$

where $\theta =(\theta^{{\text{L}}_{1}},\theta^{{\text{L}}_{2}},\theta^{{\text{L}}_{3}})$, $Y_{\text{obs}}=(Y_{\text{obs}}^{{\text{L}}_{1}},Y_{\text{obs}}^{{\text{L}}_{2}},Y_{\text{obs}}^{{\text{L}}_{3}})$ and *P*(*θ*|*Y*_obs_) is the observed-data posterior density, which is proportional to the product of a prior density *π*(*θ*) and the observed-data likelihood function given by (1.1). Our computational algorithms approximate the observed-data posterior *P*(*θ*|*Y*_obs_) as well as the posterior predictive distribution of missing data *P*(*Y*_mis_|*Y*_obs_,*θ*) by iterating between three separate Gibbs samplers, each approximating level 1, 2 and 3 observed-data posterior and posterior predictive distributions. These computational algorithms have previously been applied to a two-level model given by (2.2) to pursue inference by MI in longitudinal or clustered designs (Schafer & Yucel 2002). Variations of MCMC methods have been applied to numerous missing-data problems at level 1 (see §2*b*(iii)), which approximate the distributions $P(\theta^{{\text{L}}_{1}}|Y_{\text{obs}}^{{\text{L}}_{1}})$ and $P(Y_{\text{mis}}^{{\text{L}}_{1}}|Y_{\text{mis}}^{{\text{L}}_{1}})$ (Schafer 1997). I use these already implemented algorithms to impute missing values at levels 2 and 1 and modify the algorithm by Schafer & Yucel (2002) to draw $Y_{\text{mis}}^{{\text{L}}_{3}}$ from its posterior predictive distribution $P(Y_{\text{mis}}^{{\text{L}}_{3}}|Y_{\text{obs}}^{{\text{L}}_{3}})$ under the proposed model (2.1).

The algorithm consists of the following steps.

### (a) Step I: imputation at level 1

Impute $Y_{\text{mis}}^{{\text{L}}_{1}}$ under an appropriate imputation model as described in §2*b*(iii). Computational algorithms for creating these imputations are readily available as R packages called norm, cat or mix. Run this algorithm until convergence and keep the values of $Y_{\text{mis}}^{{\text{L}}_{1}}$.

### (b) Step II: imputation at level 2

Using the imputed values from step I, impute $Y_{\text{mis}}^{{\text{L}}_{2}}$ using the algorithm by Schafer & Yucel (2002). Here I provide a summary of this algorithm as it will be used extensively in step III. It uses a Gibbs sampler iterating between the following conditionals of the unknowns $\theta^{{\text{L}}_{2}}=(\beta ,\Sigma ,\Psi )$, $Y_{\text{mis}}^{{\text{L}}_{2}}$:

(3.2) $$b_{i}^{\left(t+1\right)}∼P(b_{i}|Y_{\text{obs}},Y_{\text{mis}}^{\left(t\right)},\theta^{(t){\text{L}}_{2}}),$$

independently for *i*=1,…,*m*; next,

(3.3) $$\theta^{(t+1){\text{L}}_{2}}∼P\left(\theta^{{\text{L}}_{2}}|Y_{\text{obs}},Y_{\text{mis}}^{\left(t\right)},B^{\left(t+1\right)}\right),$$

(3.4) $$y_{i(\text{mis})}^{(t+1){\text{L}}_{2}}∼P\left(y_{i(\text{mis})}^{(t+1){\text{L}}_{2}}|Y_{\text{obs}},B^{\left(t+1\right)},\theta^{(t+1){\text{L}}_{2}}\right),$$

where $B=(b_{1},\ldots ,b_{m})$ and $i=1,\ldots ,\sum_{i=1}^{M}\;M_{i}$. Given the starting values $\theta^{(0){\text{L}}_{2}}$ and $Y_{\text{mis}}^{(0){\text{L}}_{1}}$, these steps define one cycle of the Gibbs sampler. Executing the cycle repeatedly creates sequences $\left\{\theta^{(1){\text{L}}_{2}},\theta^{(2){\text{L}}_{2}},\ldots \right\}$ and $\left\{Y_{\text{mis}}^{(1){\text{L}}_{2}},Y_{\text{mis}}^{(2){\text{L}}_{2}},\ldots \right\}$ whose limiting distributions are $P(\theta^{{\text{L}}_{2}}|Y_{\text{obs}}^{{\text{L}}_{2}})$ and $P(Y_{\text{mis}}^{{\text{L}}_{2}}|Y_{\text{obs}}^{{\text{L}}_{2}})$, respectively. With the priors given in §2*b*(iv), each of steps (3.2)–(3.4) is derived by straightforward application of Bayes’ theorem and is described in detail by Schafer & Yucel (2002). These computations are implemented in an R package called pan. Pan is run until convergence to the distributions stated above and the values of $Y_{\text{mis}}^{{\text{L}}_{2}}$ are stored.

### (c) Step III: imputation at level 3

This step will also require a separate Gibbs sampler using quantities from steps I and II. I assume that imputed level 1 and 2 values are included in $X_{ij}$. The Gibbs sampler of step II can easily be modified for step III to create imputations under model (2.1). Note that for fixed values of *b*_*i*_ (i.e. for known values of level 1 random effects)

$$y_{ij}^{{\text{L}}_{3}*}=X_{ij}\beta +W_{ij}c_{ij}+\epsilon_{ij},$$

where $y_{ij}^{{\text{L}}_{3}*}=y_{ij}^{{\text{L}}_{3}}-Z_{ij}b_{i}$, which has the form of the simpler model (2.2). Similarly, for fixed values of *c*_*ij*_, the model reduces to

$$y_{ij}^{{\text{L}}_{3}**}=X_{ij}\beta +Z_{ij}b_{i}+\epsilon_{ij},$$

where $y_{ij}^{{\text{L}}_{3}**}=y_{ij}^{{\text{L}}_{3}}-W_{ij}c_{ij}$. Both of these conditional models are identical in form to the two-level model. It now becomes a straightforward task to construct a Gibbs sampler in the following form:

(3.5) $$b_{i}^{\left(t+1\right)}∼P(b_{i}|Y_{\text{obs}}^{{\text{L}}_{3}},Y_{\text{mis}}^{(t){\text{L}}_{3}},\theta^{(t){\text{L}}_{3}},c_{ij}^{\left(t\right)}),\;i=1,\ldots ,m;$$

(3.6) $$c_{ij}^{\left(t+1\right)}∼P(b_{i}|Y_{\text{obs}}^{{\text{L}}_{3}},Y_{\text{mis}}^{(t){\text{L}}_{3}},\theta^{\left(t\right)},b_{i}^{\left(t+1\right)}),\;i=1,\ldots ,m,\;j=1,\ldots ,m_{i};$$

(3.7) $$\theta^{(t+1){\text{L}}_{3}}∼P(\theta^{{\text{L}}_{3}}|Y_{\text{obs}}^{{\text{L}}_{3}},Y_{\text{mis}}^{(t){\text{L}}_{3}},B^{\left(t+1\right)},C^{\left(t+1\right)});$$

(3.8) $$Y_{\text{mis}}^{(t+1){\text{L}}_{3}}∼P(Y_{\text{mis}}^{{\text{L}}_{3}}|Y_{\text{obs}}^{{\text{L}}_{3}},B^{\left(t+1\right)},C^{\left(t+1\right)},\theta^{(t+1){\text{L}}_{3}}).$$

Here θ=(β,Σ,Ψ,Γ); $B={\left(\text{vec}(b_{1}),\ldots ,\text{vec}(b_{m})\right)}^{T}$; $C={\left(\text{vec}(c_{11}),\ldots ,\text{vec}(c_{m,n_{m}})\right)}^{T}$; $Y_{\text{obs}}^{{\text{L}}_{3}}=(y_{11(\text{obs})}^{{\text{L}}_{3}},\ldots ,y_{m,m_{m}(\text{obs})}^{{\text{L}}_{3}})$; and $Y_{\text{mis}}^{{\text{L}}_{3}}=(y_{11(\text{mis})}^{{\text{L}}_{3}},\ldots ,y_{m,n_{m}(\text{mis})}^{{\text{L}}_{3}})$. Given the starting values $\theta^{(0){\text{L}}_{3}}$ and $Y_{\text{mis}}^{(0){\text{L}}_{3}}$, these four steps define a Gibbs sampler in which the sequences $\left\{\theta^{(t){\text{L}}_{3}}\right\}$ and $\left\{Y_{\text{mis}}^{(t){\text{L}}_{3}}\right\}$ converge in distribution to $P(\theta^{{\text{L}}_{3}}|Y_{\text{obs}}^{{\text{L}}_{3}})$ and $P(Y_{\text{mis}}^{{\text{L}}_{3}}|Y_{\text{obs}}^{{\text{L}}_{3}})$, respectively.

There are several modifications to the Gibbs sampler (3.2)–(3.4) for a two-level model to implement (3.5)–(3.8). The prior distribution remains the same for *β*, *Σ* and *Ψ*, but now we need to add a prior distribution for the new variance component *Γ*. Following the same practice as before, we impose an inverted Wishart distribution on $\Gamma^{-1}∼W(\zeta ,ϒ)$, which exists provided that ϒ>0 and $\zeta \geq q_{2}r$. Hyperparameters can be determined in the same fashion as before, that is, $\zeta^{\left(-1\right)}{ϒ}^{\left(-1\right)}$ is a prior guess for *Γ*. Full conditionals to carry out the Gibbs sampler defined by (3.5)–(3.8) are given in appendix A.

Determining the values of hyperparameters on the priors for the variance components is an important practical task. For the level 3 model (2.1), excellent prior guesses for *Σ*, *Ψ* and *Γ* may be obtained by temporarily supposing that *Σ* is diagonal, and *Ψ* and *Γ* are block diagonal. Under these conditions, the multivariate model separates into independent univariate models for each of the *r* columns of *y*_*ij*_, and maximum-likelihood estimates of the variance components may be quickly calculated using existing software for linear mixed-effects models. Determining the starting values for steps I and II is easier as model-fitting techniques for level 2 model (2.2) or for level 1 are directly available (Schafer 1997; Yucel 2007).

Iterating steps I–III for predetermined cycles for number of cycles *K* will result in *K* ‘completed’ datasets. The algorithm given above saves imputations from a Gibbs sampler upon convergence. It is possible to view each of steps I–III as a Gibbs step and running it until convergence would roughly give similar results. In the latter, one would have to monitor convergence (in distribution) to $P(Y_{\text{mis}}|Y_{\text{obs}},\theta )$ and $P(\theta |Y_{\text{obs}})$. Running each of the Gibbs samplers given in steps I–III, on the other hand, would require monitoring convergence to $P(Y_{\text{mis}}^{{\text{L}}_{i}}|Y_{\text{obs}}^{{\text{L}}_{i}},\theta^{{\text{L}}_{i}})$ and $P(\theta^{{\text{L}}_{i}}|Y_{\text{obs}}^{{\text{L}}_{i}})$ would be necessary within each step.

After imputation, the resulting *K* versions of the complete data are analysed separately by complete-data methods, and the results are combined using simple arithmetic to obtain inferences that effectively incorporate uncertainty due to missing data. As shown by Rubin (1987), quality inferences can often be obtained with a very small number (e.g. *K*=5) of imputations. Methods for combining the results of the complete-data analyses are given by Rubin (1987, 1996) and reviewed by Schafer (1997, ch. 4).

## 4. Application

The data for this example were taken from the cross-sectional National Survey for Children with Special Health Care Needs (NSCSHCN). The survey was conducted from April 2000 to October 2002, by the National Center for Health Statistics at the Centers for Disease Control and Prevention, using the State and Local Area Integrated Telephone Survey of list-assisted random digit dialling. A screening tool determined the presence of a special health care need. Final sample size used for the imputation was 36 491 children.

The substantive goal of this study was to determine the association between Medicaid-managed paediatric behavioural health care programmes, and other state-level factors, with the unmet need for mental health care among children with special health care needs (CSHCN). To study the impact of the state-level factors, data were supplemented by additional state-level sources. These sources provided data on variables such as: metropolitan statistical area, primary care paediatrician (PCP) and mental health provider (MHP) supply; the region of the country; and state Medicaid mental health programme type. Owing to lack of resources, however, information on provider supplies (which are indicator variables measuring whether a state has fewer PCP than median) was incomplete for some of the states. Many items asked in NSCSHCN were arbitrarily missing. Missing values in most items were moderate with rates changing from 0.02 to 3%. However, on items carrying great substantive importance, such as whether the respondent received all needed mental health care, 35% values were missing; poverty level indicator was missing at approximately 10%; the indicator on whether the child needed substance abuse treatment was missing at approximately 27%.

The two-level model given by (2.2) with random intercepts to account for clustering at the state level was used to impute missing values at the individual level. This model included covariates such as race, insurance type, health services and insurance service indicators, medical expenditures and state-level covariates. I also used covariates that were not necessarily meaningful for the substantive findings of the study, but were thought to be informative on describing missingness. I used a loglinear model with fully observed covariates (some of which were aggregated up to state level from individual data) to impute state-level missing values.

One of the dependent variables that carried particular importance was the unmet health care need defined as whether mental health care or counselling during the previous 12 months was needed, but was not received. A logistic regression with random intercept was estimated for each of the 10 imputed datasets, and the results indicated that living in a state with below the median per capita number of mental health providers was associated with greater unmet mental health care need, compared with having above the median ones (estimated odds ratio, OR=1.30). Among children with special health care need who have only Medicaid for insurance, living in a state with a Medicaid-managed care programme (OR=1.97) and, specifically, states in which mental and physical health services are financed separately (OR=2.00), was associated with greater unmet mental health care need compared with a fee-for-service programme. An important estimate of the impact of missing values on the standard errors as well as a diagnostic measure on how well the imputation models perform is the rate of missing information (Schafer 1997; Harel 2007). Assessing the rates of missing information on the estimated coefficients indicated that our imputation models performed well enough to reduce the rates from the observed rates of missingness.

## 5. Discussion and future research

The main goal of this paper was to provide a principled and easy-to-implement method for dealing with missing values in multilevel applications. Our method improves current techniques relying on MI by allowing missing values at any level of observational units in multilevel applications. Because our techniques rely mostly on previously implemented algorithms, they can easily be used by practitioners. Our current work is limited, however, in evaluating its sensitivity to deviations from assumptions, particularly deviations from the ignorability assumption. I believe that when the imputation models are kept as rich as possible to the extent where they are estimable, the biases due to wrongly assumed missingness mechanism are minimal. Another important violation can occur on the assumption of normality. Several studies, however, show that the inferential quality of MI under normality is acceptable even when the normality assumption is clearly violated (Demirtas *et al*. 2008).

The computational algorithms considered here are not the most efficient ones. Methods for improving the speed or convergence are available (van Dyk & Meng 2001; Gelman & Hill 2007). If any of the Gibbs steps could be carried out without conditioning on the simulated values of *Y*_mis_ or the random effects, then the algorithms could be made to converge in fewer iterations. However, we believe that if the goal is preparing multiply imputed datasets to be analysed by multiple users, the speed or efficiency of the algorithm is of little importance as the process of preparing MI is done only once. Further, with improved computing facilities, Gibbs iterations can be performed quickly even with the large datasets provided that sufficient physical memory is available to store observed and missing data as well as parameter values across the iterations.

Our methods can be extended in several directions. The first extension pertains to taking advantage of the versatility of random-effects models. Consideration of random-effects models provides great flexibility in preserving important relationships among imputed datasets. This point is very important for applications where MI is used by multiple users and hence the imputer's goal is to preserve the relationship to minimize the biases from a wide variety of analyses using the imputed data. In some applications, it may be desirable to preserve structures beyond means. Random-effects models can easily be extended to accommodate other special structures such as random covariances (Yucel 2000). In other applications, random-effects models can be used to accommodate multiple membership or ambiguous membership problems, typically seen in education and genetic epidemiology (Foulkes *et al*. in press).

The second direction pertains to handling diverse sets of incompletely observed variables. Consideration of joint models on the variables subject to missingness may not be realistic in some instances. These include surveys with items that are only applicable to subpopulations (e.g. item asking respondents when was the last time they had a pap smear or how many cigarettes they smoked during the last week) or items with skip patterns. Further, most surveys or data systems contain incompletely observed items measured on different scales. Imposing a joint distribution may not be feasible as such a joint distribution may not even exist. When the items are all ordinal or binary, approximations are acceptable, but when they have nominal, count or semi-continuous items, then the joint modelling strategy may not work. Several studies focus on drawing from pragmatic conditional distributions which may not define a joint distribution in the way the conditionals of Gibbs samplers define (Van Buuren & Oudshoorn 2000; Raghunathan *et al*. 2001). Such algorithms lead to ‘improper’ imputations (Rubin 1987) but may be the only practical way to create MI. Our current work is on extending these methods to multilevel applications. Some of the initial results appeared in Yucel & Raghunathan (2006).

Another important extension is to incorporate different missingness mechanisms. In some applications, especially in longitudinal datasets resulting from clinical trials, data may be missing under a non-ignorable mechanism. While estimation techniques under non-ignorable missingness are available, they tend to be problem specific and sensitive to departures from assumptions (Demirtas & Schafer 2003; Demirtas 2005). Developing MI under non-ignorable models and investigating the sensitivity of MI inference to a misspecified missingness mechanism are important topics to study. Another important research topic is the incorporation of different types of missingness mechanisms. It is possible that two different mechanisms can coexist in a given application. Harel (2007) develops inferences using two-stage MI, which can be useful to extend in multilevel data applications.

## Footnotes

Observed likelihood (1.1) is generally induced by a model rich enough to contain the model of the subject matter.

## Appendix A

Below I provide the full conditionals of each of steps (3.5)–(3.8). I omit technical details, which can be provided upon request. Drawing level 2 random effects, *b*_*i*_ is virtually the same as step (3.2) of the Gibbs sampler for the two-level model

$$\text{vec}(y_{ij}^{**{\text{L}}_{3}})|b_{i},c_{ij},\theta ∼N(\text{vec}(X_{ij}\beta +Z_{ij}b_{i}),(\Sigma ⊗I_{n_{ij}})),$$

$$\text{vec}(b_{i})|\theta ∼N(0,\Psi ),$$

independently for i=1,…,M. Once level 2 random effects, {*c*_*ij*_}, are known then we can treat all cases within level 1 units as independent. It follows that:

$$\text{vec}(b_{i})|y_{ij}^{**},\theta ∼N(\text{vec}({\tilde{b}}_{i}),U_{i}),$$

where

(A1) $$\text{vec}({\tilde{b}}_{i})=U_{i}(\Sigma^{-1}⊗Z_{ij}^{T})\text{vec}(y_{ij}^{**}-X_{ij}\beta ),$$

(A2) $$U_{i}={\left(\Psi^{-1}+(\Sigma^{-1}⊗Z_{ij}^{T}Z_{ij})\right)}^{-1}.$$

Similarly, for fixed *b*_*i*_, the posterior distribution of *c*_*ij*_ is

$$\text{vec}(c_{ij})|y_{ij}^{*},\theta ∼N(\text{vec}({\tilde{c}}_{ij}),V_{ij}),$$

where

(A3) $$\text{vec}({\tilde{c}}_{ij})=V_{ij}(\Sigma^{-1}⊗W_{ij}^{T})\text{vec}(y_{ij}^{*}-X_{ij}\beta ),$$

(A4) $$V_{ij}={\left(\Gamma^{-1}+(\Sigma^{-1}⊗W_{ij}^{T}W_{ij})\right)}^{-1}.$$

Simulation of *θ* in (3.7) proceeds as follows: *Ψ*^−1^ is drawn from its posterior $W(\nu_{2}^{\prime },\Lambda_{2}^{\prime })$, where $\nu_{2}^{\prime }=\nu_{2}+\sum_{i=1}^{M}\;M_{i}$ and $\Lambda_{2}^{\prime }={\left(\Lambda +B^{T}B\right)}^{-1}$. Similarly, $\Gamma^{-1}$ is simulated from a Wishart distribution $W(\zeta^{\prime },{ϒ}^{\prime })$, where $\zeta^{\prime }=\zeta +\sum_{i=1}^{m}\;m_{i}$ and ${ϒ}^{\prime }={\left({ϒ}^{-1}+C^{T}C\right)}^{-1}$. Next, calculate the ordinary least-squares coefficients

$$\hat{\beta }={\left(\sum_{i=1}^{m}\sum_{j=1}^{m_{i}}X_{ij}^{T}X_{ij}\right)}^{-1}\left(\sum_{i=1}^{m}\sum_{j=1}^{m_{i}}X_{ij}^{T}(y_{ij}-Z_{ij}b_{i}-W_{ij}c_{ij})\right),$$

and residuals ${\hat{\epsilon }}_{ij}=y_{ij}-X_{ij}\hat{\beta }-Z_{ij}b_{i}-W_{ij}c_{ij}$, and draw *Σ*^−1^ from a Wishart distribution with degrees of freedom $\nu_{1}^{\prime }=\nu_{1}-p+\sum_{i=1}^{m}\;\sum_{j=1}^{m_{i}}\;n_{ij}$ and scale matrix $\Lambda_{1}^{\prime }={\left(\Lambda_{1}^{-1}+\sum_{i=1}^{m}\sum_{j=1}^{m_{i}}{\hat{\epsilon }}_{ij}^{T}{\hat{\epsilon }}_{ij}\right)}^{-1}$. Finally, draw *β* from a multivariate normal distribution centred at $\hat{\beta }$ with covariance matrix Σ⊗V, where $V={\left(\sum_{i=1}^{m}\sum_{j=1}^{m_{i}}X_{ij}^{T}X_{ij}\right)}^{-1}$.

To carry out the final step (3.8) of the Gibbs sampler, note that the rows of $\epsilon_{ij}=y_{i}-X_{i}\beta -Z_{i}b_{i}$ are independent and normally distributed with mean zero and covariance matrix *Σ*. Therefore, in any row of *ϵ*_*ij*_, the missing elements have an intercept-free multivariate normal regression on the observed elements; the slopes and residual covariances for this regression can be quickly calculated by inverting the square submatrix of *Σ* corresponding to the observed variables. Drawing the missing elements in *ϵ*_*ij*_ from these regressions and adding them to the corresponding elements of $X_{i}\beta +Z_{i}b_{i}$ complete the simulation of $y_{(\text{mis})ij}^{{\text{L}}_{3}}$.
